# A novel DNA methylation signature is associated with androgen receptor activity and patient prognosis in bone metastatic prostate cancer

**DOI:** 10.1186/s13148-021-01119-0

**Published:** 2021-06-30

**Authors:** Erik Bovinder Ylitalo, Elin Thysell, Mattias Landfors, Maria Brattsand, Emma Jernberg, Sead Crnalic, Anders Widmark, Magnus Hultdin, Anders Bergh, Sofie Degerman, Pernilla Wikström

**Affiliations:** 1 Department of Medical Biosciences, Pathology, Umeå University, Umeå, Sweden; 2 Department of Surgical and Perioperative Sciences, Orthopedics, Umeå University, Umeå, Sweden; 3 Department of Radiation Sciences, Oncology, Umeå University, Umeå, Sweden; 4 Department of Clinical Microbiology, Umeå University, Umeå, Sweden

**Keywords:** Prostate cancer, Metastasis, Subtypes, MetA, MetB, MetC, DNA methylation, Gene expression, Androgen receptor, Prognosis

## Abstract

**Background:**

Patients with metastatic prostate cancer (PC) are treated with androgen deprivation therapy (ADT) that initially reduces metastasis growth, but after some time lethal castration-resistant PC (CRPC) develops. A better understanding of the tumor biology in bone metastases is needed to guide further treatment developments. Subgroups of PC bone metastases based on transcriptome profiling have been previously identified by our research team, and specifically, heterogeneities related to androgen receptor (AR) activity have been described. Epigenetic alterations during PC progression remain elusive and this study aims to explore promoter gene methylation signatures in relation to gene expression and tumor AR activity.

**Materials and methods:**

Genome-wide promoter-associated CpG methylation signatures of a total of 94 tumor samples, including paired non-malignant and malignant primary tumor areas originating from radical prostatectomy samples (*n* = 12), and bone metastasis samples of separate patients with hormone-naive (*n* = 14), short-term castrated (*n* = 4) or CRPC (*n* = 52) disease were analyzed using the Infinium Methylation EPIC arrays, along with gene expression analysis by Illumina Bead Chip arrays (*n* = 90). AR activity was defined from expression levels of genes associated with canonical AR activity.

**Results:**

Integrated epigenome and transcriptome analysis identified pronounced hypermethylation in malignant compared to non-malignant areas of localized prostate tumors. Metastases showed an overall hypomethylation in relation to primary PC, including CpGs in the *AR* promoter accompanied with induction of *AR* mRNA levels. We identified a Methylation Classifier for Androgen receptor activity (MCA) signature, which separated metastases into two clusters (MCA positive/negative) related to tumor characteristics and patient prognosis. The MCA positive metastases showed low methylation levels of genes associated with canonical AR signaling and patients had a more favorable prognosis after ADT. In contrast, MCA negative patients had low AR activity associated with hypermethylation of AR-associated genes, and a worse prognosis after ADT.

**Conclusions:**

A promoter methylation signature classifies PC bone metastases into two groups and predicts tumor AR activity and patient prognosis after ADT. The explanation for the methylation diversities observed during PC progression and their biological and clinical relevance need further exploration.

**Supplementary Information:**

The online version contains supplementary material available at 10.1186/s13148-021-01119-0.

## Introduction

Prostate cancer (PC) is a very common malignancy and a major cause of cancer mortality in men worldwide (https://gco.iarc.fr). Most patients with lethal PC develop bone metastases, which are treated with androgen deprivation therapy (ADT). Initially, ADT reduces metastasis growth, but eventually castration-resistant PC (CRPC) develops. Several treatment strategies for metastatic CRPC exist, with the majority aiming at inhibiting androgen receptor (AR) signaling [1]*.* Patients with CRPC show diverse responses to AR-inhibiting drugs, ranging from a strong response to complete resistance, underlining the need for complementary/alternative treatments. To guide future therapeutic developments, the tumor biology of metastatic PC must be understood in more detail.

The genetics behind CRPC have been comprehensively explored [2–5], indicating *AR* amplification as the most common event (seen in about 50% of the cases). Also, structural *AR* rearrangements and *AR* mutations are frequently observed together with mutations in co-factors regulating the transcriptional activity of the AR. The prognostic and therapy-predicting value of those genetic defects in relation to AR targeting therapies is still unclear [5, 6].

By exploring the gene expression in clinical samples of PC bone metastases, we have identified transcriptomic as well as proteomic profiles of suggested clinical relevance [7–12]. Specifically, we have identified three molecular subtypes of bone metastases, termed MetA–C, based on the diverged expression of genes related to AR activity, cell cycle activity, and stroma response [12]. The MetA subtype shows high AR activity in comparison to MetB and C, while MetB shows higher cell cycle activity than the other subtypes. Accordingly, MetA patients have the best prognosis after ADT, while MetB patients have by far the worst prognosis. Interestingly, the MetA–C subtypes could be intrinsic, as selected features of MetB (low AR activity and high proliferation) were traceable back to the corresponding primary tumors [12]. In line with this, PC subtypes with characteristics similar to the MetA–C subtypes have been identified by transcriptomic analysis of primary prostate tumors [13, 14].

The mechanistic explanation for the development of different metastasis subtypes is unknown. The subtypes could relate to diverse basal/luminal cellular origin, genetic defects as described above, but also to epigenetic defects affecting differentiation and clonal expansion, possibly influenced by the metastatic microenvironment and/or by therapy. Alterations in DNA methylation have been described to contribute to PC development and progression into metastatic disease [15–17]. The aim of the current study was to explore the promoter DNA methylation pattern in relation to gene expression during PC disease progression. Specific interest was put into analyzing promoter methylation levels in relation to canonical AR activity in metastasis samples and to patient prognosis.

## Material and methods

### Patients

Samples of bone metastases were obtained from a series of fresh-frozen biopsies collected from 70 patients with PC operated for metastatic spinal cord compression at Umeå University Hospital (2003–2013). The patient series have been previously described [7, 9, 12] and clinical characteristics of patients included in the current study are summarized in Table 1. The study also included 12 separate patients with localized PC who were treated with radical prostatectomy at Umeå University Hospital, between 2008 and 2009; mean age for these men was 60 years (range 48–68 years) and mean serum level of prostate specific antigen (PSA) was 11 ng/ml (range 3.5–26 ng/ml). Clinical local stage was T2 (*n* = 4) or T3 (*n* = 8) and Gleason score (GS) was 7 (*n* = 10) or 8 (*n* = 2).

**Table 1:** Clinical characteristics of patients with prostate cancer from whom samples of bone metastases were analyzed by Infinium Methylation EPIC arrays

Clinical characteristics	Hormone-naíve *n* = 14	Short-term castrated^a^ *n* = 4	Castration-resistant^b^ *n* = 52
Age at diagnosis (years)	78 (74–80)	75 (72–76)	71 (64–75)
Age at metastasis surgery (years)	–	–	73 (68–80)
Serum PSA at diagnosis (ng/ml)	320 (82–980)	2300 (560–4300)	82 (40–510)
Serum PSA at metastasis surgery (ng/ml)	–	–	250 (82–630)
Bicalutamide prior to metastasis surgery^c^			
Yes	0	0	28
No	14	4	24
Radiation prior to metastasis surgery^d^			
Yes	0	0	7
No	14	4	45
Chemotherapy prior to metastasis surgery			
Yes	0	0	9
No	14	4	43
Ra-223 prior to metastasis surgery			
Yes	0	0	6
No	14	4	46

Continuous values are given as median (25th; 75th percentiles)

^a^Short-term treated patients had received androgen ablation therapy for 1–3 days before metastasis surgery

^b^Castration-resistant patients had disease progression after long-term androgen deprivation therapy. First line androgen-deprivation therapy (ADT) includes surgical ablation, LHRH/GNRH agonist therapy, and therapy with anti-androgens

^c^Castration therapy as stated above and bicalutamide for treatment of CRPC

^d^Radiation towards operation site

### Tissue sample preparation

Bone metastasis samples were instantly fresh-frozen in liquid nitrogen. Fresh radical prostatectomy specimens were received at the pathology department immediately after surgery and cut into 0.5 cm thick slices. From these slices, 20 samples were taken using a 0.5 cm skin punch and frozen in liquid nitrogen within 30 min after surgery. The prostate slices were formalin-fixed, embedded in paraffin, cut in 5 µm sections, whole-mounted, and stained with hematoxylin–eosin. Tissue sample composition (non-malignant or malignant) of the frozen pieces was determined from their location in the whole-mount sections**.**

Representative areas of fresh-frozen bone metastasis (M) samples, non-malignant (N) and malignant tumor (T) areas, and prostatectomy tissue samples were cryo-sectioned into extraction tubes. The fraction of epithelial cells in the samples was determined by examination of parallel hematoxylin–eosin-stained sections.

### DNA methylation profiling

Genomic DNA was isolated by the AllPrep DNA/RNA/Protein method (Qiagen, Hilden, Germany) and DNA quality and quantity were determined by spectrophotometry (NanoDrop, Thermo Scientific, Wilmington, DE, USA) and the Qubit dsDNA BR assay kit on a Qubit 3.0 Fluorometer (Invitrogen, Carlsbad, CA). DNA (300 ng) was bisulfite converted using the EZ DNA Methylation Kit (Zymo Research, Irvine, USA) and thereafter applied to the Infinium Methylation EPIC arrays (lllumina, San Diego, CA), and operated according to the manufacturer’s instructions. Array analysis including pre-processing and normalization was performed as previously described [18], with some modifications. The quality of each array was evaluated with built-in controls and the matching identities of the N and T paired samples were confirmed by using the 59 built-in single nucleotide polymorphisms (SNPs) (Additional file 1: Fig. S1).

The fluorescence intensities were extracted using the Methylation Module (1.9.0) in the Genome Studio software (V2011.1), whereas pre-processing and downstream analysis was done using R (v3.4.1). An overview of the pre-processing steps is shown in Additional file 2: Fig. S2. Data was normalized using the BIMQ method to compensate for the two different bead types used in the array [19]. Cross-reactive CpG probes that aligned to multiple loci in the genome or were located in methylation quantitative trait loci (meQTLs) [20, 21] or located less than 5 bp from a known single nucleotide polymorphism in the European population [22] were excluded. CpG probes with detection *P* value > 0.05 in any sample were also excluded. The methylation level (*β*-value) of each CpG site ranging from 0 (no methylation) to 1 (complete methylation) was used as the measure for methylation level in down-stream analyses.

Methylation levels (*β*-values) were extracted for promoter associated CpGs located in the TSS1500 (the region that covers − 200 to − 1500 nucleotides upstream of Transcription Start Site (TSS)), TSS200 (from TSS to − 200 nucleotides upstream of TSS), and 5′UTR regions, which showed an overall SD > 0.05. Differentially methylated CpG sites (DM-CpGs) were defined as a mean delta-*β*-value > 0.3 or <  − 0.3 between compared groups. Heatmaps were produced in R (v3.4.1) without scaling and using default settings for clustering.

Principal component analysis (PCA), based on centralized *β*-values were used for unsupervised multivariate projection.

### Copy number variation analysis

The raw signal intensity data from the HumanMethylationEPIC arrays was imported to R by the minfi package [23] and CNV analysis was performed using the conumee package [24]. The 12 non-malignant samples were used as reference samples and the analysis included the *AR* gene as a detailed region. The CNV status of AR was determined through manual inspection. Human genome GRCh37 (NCBI)/hg19 was used for assigning all chromosome positions.

### Whole genome expression analysis

The majority (96%) of the samples applied to DNA methylation analysis had been previously applied to whole genome expression array analysis using the human HT12 Illumina Beadchip technique (Illumina, San Diego, CA) [7, 9]. In 48 cases, total RNA was extracted in parallel with the DNA extractions using the AllPrep DNA/RNA/Protein method. In the other cases, RNA had been extracted from parallel tissue sections using the AllPrep or the Trizol method (Invitrogen, Carlsbad, CA). Bead chip data from two separate gene expression studies (GEO Datasets GSE29650 and GSE101607) were combined for all probes with average signals above two-times the mean background level in at least one sample per study array, leaving 15,232 gene transcripts for subsequent analysis. The gene expression arrays were individually normalized using the quantile method and data was centered by the mean for each probe. Gene transcripts were matched to corresponding promoter CpG sites by Refseq accession and Entrez gene identification numbers.

### Assessment of AR activity and proliferation in metastasis samples

The AR activity of a metastasis sample was defined by its relative expression levels of genes predefined to be associated with canonical AR activity; *AR, FOXA1, HOXB13, KLK2, KLK3, NKX3-1, STEAP2,* and *TMPRSS2,* as previously described [9]. The AR activity of each metastasis sample was represented by its score on the first PCA score vector (*t*[1]), capturing the largest variation in the data as a linear combination of the selected transcript levels. Similarly, tumor cell proliferation in metastasis samples was represented by the *t*[1] scores obtained from PCA of genes predefined to be associated with cell cycle proliferation [25]; *RAD54L, CDC20, CENPF, CDKN3, PBK, TOP2A, LCMT2, ASF1B, KIF20A, CDCA8, NUSAP1, PRC1, PLK1, CDCA3, CEP55, CDC2, KIF11, BUB1B, TK1, ASPM, PTTG1, ORC6L, FOXM1, RAD51, CENPM, CDAN1, KIAA0101, MCM10****.***

### Classification of metastasis samples into metastasis subtypes

The metastasis samples were classified into molecular metastasis subtypes MetA (*n* = 46), MetB (*n* = 12), and MetC (*n* = 8) by PCA and unsupervised clustering based on their transcription profiles, as previously described [12]. Four samples were not possibly to classify in relation to MetA–C, due to lack of transcriptomic data.

### Functional enrichment analysis

Functional enrichment analysis was performed by the MetaCore software (GeneGo, Thomson Reuters, New York, NY). In comparisons between different groups (T and N or M and T), analysis was based on genes showing (i) DM-CpGs (mean delta-*β*-value > 0.3 or <  − 0.3 in promoter regions), (ii) inverse correlation between the methylation levels (*β* values) of CpG sites (mCpGs) and the corresponding transcript levels, and iii) significantly different transcript levels (*P* < 0.05). In the analysis of promoter methylation levels in relation to AR activity, analysis was based on genes showing (i) positive or negative correlations between mCpGs (*β* values) and AR activity score (corr > 0.4; −  < 0.4), (ii) a considerable difference between samples (stdev > 0.15), and (iii) inverse correlation (> 0.4) between mCpGs (*β* values) and the corresponding transcript levels. Sets of genes associated with a functional pathway were determined as significantly enriched based on *P* values representing the probability for a process to arise by chance, considering the numbers of enriched gene products in the data versus the number of genes in the process. *P* values were adjusted by considering the rank of the process, given the total number of processes in the MetaCore ontology. Enriched pathways were expressed as pathway maps or as process networks, which were created by MetaCore on the basis of both pathway maps and gene ontology processes.

### Statistics

The Wilcoxon rank sum test was used to compare independent groups and the Wilcoxon signed rank sum test for paired data. Bivariate correlations were analyzed according to Pearson. Survival analysis was performed by Kaplan–Meier analysis and by multivariate Cox regression analysis, with death of PC as event and death by other causes as censored events. The false discovery rate (FDR) were controlled according to the Benjamini–Hochberg procedure. To evaluate the consistency of the clusters a silhouette analysis was performed using the cluster package [26] in R.

## Results

### DNA promoter methylation associated with prostate cancer development and progression

The methylation levels (*β*-values) of CpG sites (mCpGs) were analyzed in pairs of non-malignant (N) and malignant primary tumor (T) areas originating from radical prostatectomy samples of 12 PC patients. In parallel, the mCpG profiles were analyzed in 70 bone metastasis (M) samples of separate patients with hormone-naive (HN, *n* = 14), short-term castrated (ST, *n* = 4), or castrate-resistant disease (CRPC, *n* = 52) (Table 1). To facilitate correlation studies between methylation levels of gene promoter regions and corresponding transcript levels, the analysis was focused on CpG sites in promoter regions (defined as CpGs located within the TSS1500, TSS200, 5´UTR regions), leaving 121,944 sites for down-stream analysis after filtration (Additional file 2: Fig. S2).

**Fig. 1:**
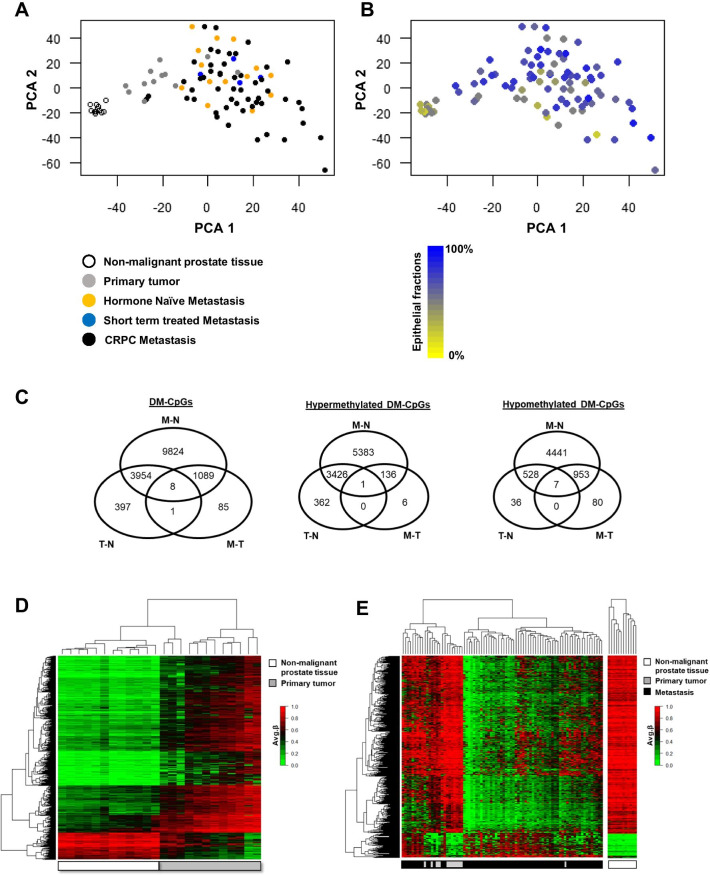
Identification of differently methylated CpGs (DM-CpGs) in non-malignant prostate (N), primary prostate tumor (T), and bone metastasis (M) tissue samples. **A**, **B** PCA plots based on 121,944 promoter associated CpGs in the HumanMethylation EPIC arrays. **A** The colors represent patient treatment; no treatment/hormone naïve (orange), castrate-resistant prostate cancer (CRPC) (black), and short term androgen deprivation therapy (blue). Non-malignant (white) and primary tumor (grey) tissues are shown. **B** The same loading plot as in Fig. **A** with epithelial fractions shown. **C** DM-CpG sites between the primary tumor (T) and non-malignant (N) prostate tissue (*n* = 4360), between the N and metastasis (M) tissue (*n* = 14,875) and between the M and T tissue (*n* = 1183). The overlaps include DM-CpGs both in the same direction between comparisons (e.g. hyper-hyper) as well as DM-CpGs in different directions (e.g. hypo-hyper). The hyper- and hypomethylated CpGs are also separately shown. **D** Heatmap showing the 4360 DM-CpGs (identified in Fig. **C**) between T (*n* = 12) and N (*n* = 12). **E** Heatmap showing the 1183 DM-CpGs (identified in Fig. **C**) between M (*n* = 70) and T (*n* = 12)

Principal component analysis based on the 121,944 promoter-associated mCpGs showed a clear separation between the N, T, and M samples, except for two T and two M samples (Fig. 1A). By histological re-examination of tissue sections, the miss-classified samples could not be explained by any obvious morphological characteristics.

The epithelial cell fraction was estimated by microscopy examination of tissue sections. The N samples showed a significantly lower epithelial cell content compared to the T and M samples; median (25th; 75th percentiles) epithelial fraction in N, T, and M samples were 50 (30–50), 80 (70–80), and 70 (50–80)%, respectively (*P* < 0.001, Fig. 1B). The M samples did not cluster related to epithelial cell fraction (Fig. 1B) or previous treatment (Fig. 1A). Based on a difference in *β*-value of at least ± 0.3, a total of 15,358 DM-CpGs were found for T, N, and/or M samples (Fig. 1C).

Differential methylation analysis between T and N samples resulted in 4360 DM-CpGs, most of which (86%) corresponding to CpG hypermethylation of the T samples (Fig. 1C). Those DM-CpGs (representing 1759 unique genes) clearly separated T and N samples in a hierarchical cluster analysis (Fig. 1D). The pronounced hypermethylation seen in the tumor samples was associated with significantly reduced gene expression levels in 25% (*n* = 442) of the DM genes (Additional file 4: Table S1). Functional enrichment analysis indicated altered processes of potential importance for cellular transformation and cancer development, including e.g. regulation of proliferation, angiogenesis and cell adhesion (Additional file 5: Table S2). In contrast, genes showing hypomethylation in combination with significantly induced expression in primary PC (*n* = 67, 4% of the DM genes) (Additional file 4: Table S1) showed no significant functional enrichment, according to the MetaCore software (Additional file 5: Table S2).

To further focus on DNA methylation alterations associated with progression from a primary tumor in the prostate to metastasis in the bone, differently methylated genes between these stages were identified. Among the 1183 DM-CpGs between T and M, the majority (88%) corresponded to hypomethylation of the M samples (Fig. 1C, E). Notably, only 70 of the hypomethylated genes also showed significantly increased transcript levels and for these no consensus regarding enriched ontologies could be found, while functional enrichment analysis of the few hypermethylated genes with parallel reduction in gene expression (*n* = 23) suggested altered apoptosis response in metastases compared to primary tumors (Additional file 5: Table S2 and Additional file 6: Table S3).

**Fig. 2:**
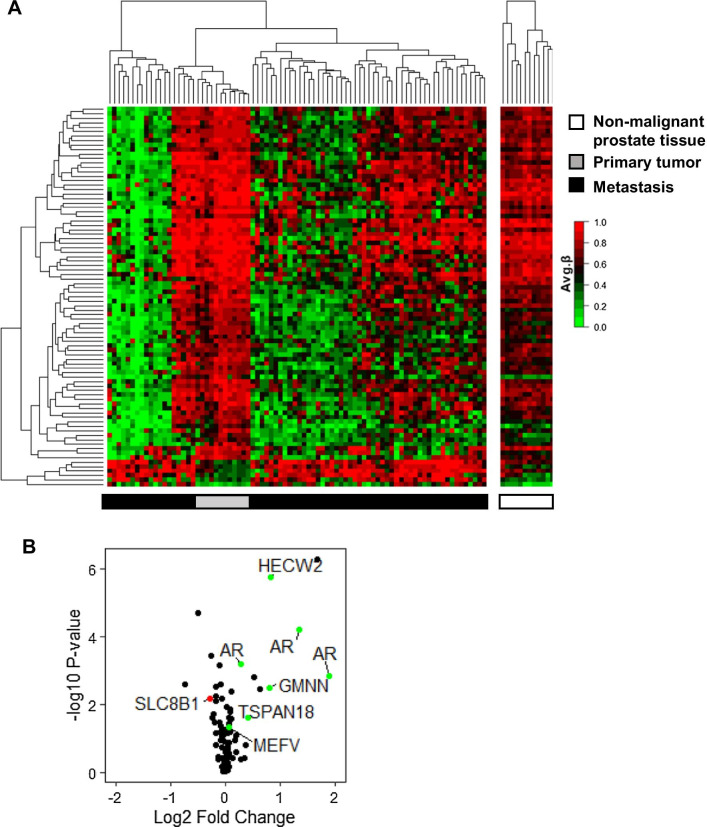
Integrated analysis of promoter DNA methylation and gene expression in metastases and primary tumors. **A** Heatmap showing the 85 unique differently methylated CpGs (identified in Fig. 1C) between the primary tumors (T) (*n* = 12) and the bone metastases (M) (*n* = 70). **B** Vulcano plot of the corresponding gene expression. 6 unique genes that were either hypermethylated with decreased gene expression (red) or hypomethylated with increased gene expression (green) in metastasis compared to primary tumors are indicated. The *AR* is represented by 3 different gene probes in the Illumina BeadChip array data, corresponding to two *AR* transcripts (coding for the full-length AR and the AR45 splice variant)

The fact that most of the DM-CpGs seen between M and T were also DM between M and N, suggests that the overlapping CpGs were likely organ-related rather than associated with bone metastasis (Fig. 1C). Further analysis was therefore focused on the 85 DM-CpGs (80 unique genes) that uniquely diverged between M and T and possibly related specifically to disease progression (Figs. 1C, 2A). Of the 85 DM-CpGs between M and T samples, 94% showed a general hypomethylation in the metastases (Figs. 1C, 2A). Hierarchical cluster analysis identified three main clusters with diversity in the degree of hypermethylation among the M samples (Fig. 2A). These clusters did not correlate to treatment status, nor to epithelial cell fraction (data not shown). Next, an integrated transcriptome analysis that aimed to identifying hyper- or hypomethylated genes with differential gene expression of potential relevance for bone metastasis was performed. Corresponding transcript levels were available for 55 of the 80 unique differently methylated genes. A set of genes showing either hypermethylation in combination with significant gene expression downregulation (*SLC8B1*) or hypomethylation in combination with gene overexpression (*HECW2, AR, GMNN, TSPAN18, and MEFV*) in metastases (Fig. 2B) were identified. The hypomethylated ubiquitin ligase *HECW2*, the DNA replication inhibitor *GMNN* (geminin), and the tetraspanin *TSPAN18* showed clear overexpression in metastases, and may deserve further studies, while fold changes for the innate immunity regulator *MEFV* and the sodium/calcium exchanger *SLC8B1* were less convincing. Of special notice was the hypomethylation of the *AR* gene in the M samples. Although gene amplification and overexpression of the *AR* in CRPC have been thoroughly described in the literature, less is known about its epigenetic regulation, and the *AR* was therefore chosen for further exploration.

### Androgen receptor gene amplification, promoter DNA hypomethylation, and mRNA overexpression in metastases

**Fig. 3:**
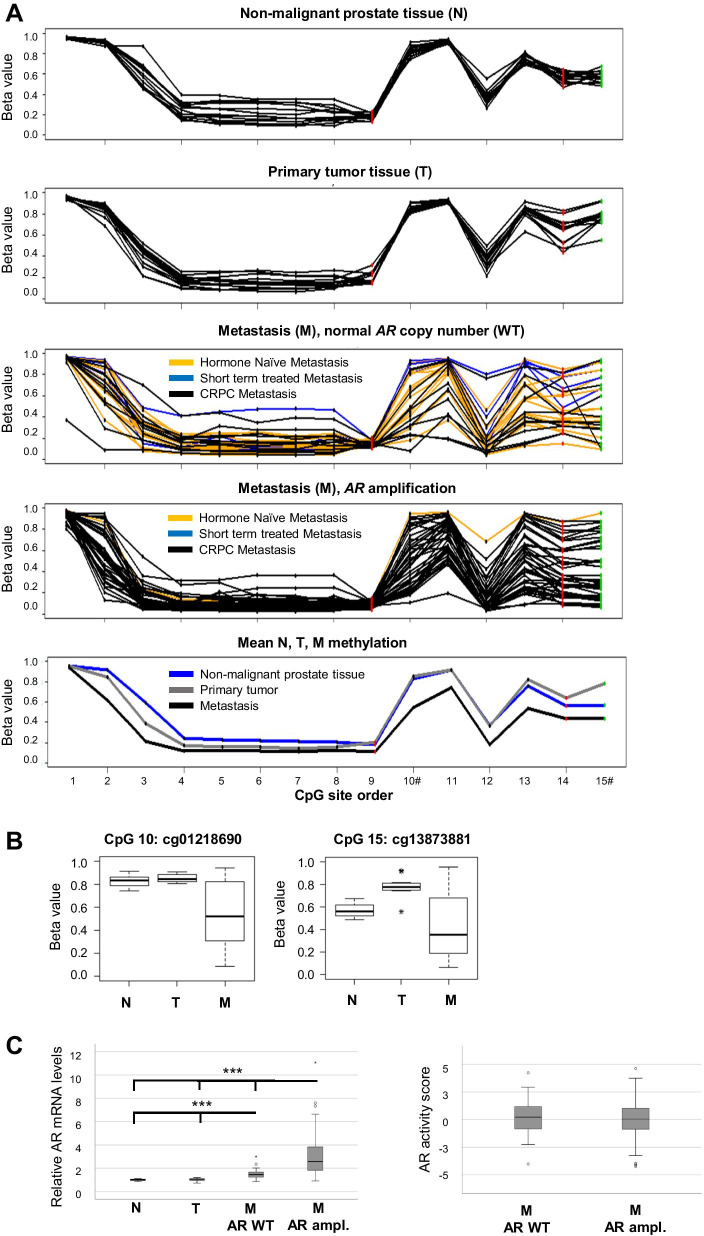
Promoter DNA methylation and gene expression of the androgen receptor (AR) during prostate cancer disease progression. **A** Methylation beta levels at individual CpGs in the *AR* promoter region for non-malignant (N, *n* = 12), primary tumor (T, *n* = 12) and metastasis samples with (M, *n* = 42) and without (*n* = 24) *AR* amplification, as well as mean N/T/M methylation levels. *AR* amplification status was defined according to copy number analysis performed based on signal intensity data from the HumanMethylationEPIC arrays, as described in the method section. Each CpG is shown as a dot, and specified in Additional file 7: Table S4. Red dots represent CpGs within the TSS200 region close to the two alternative transcription start sites, black dots represent CpGs within the TSS1500 promoter region and the green dots CpGs within the 5′UTR region. **B** Box plots of mean methylation beta values in the N/T/M group of the two differently methylated CpG (DM-CpG) sites (Illumina ID cg01218690 and cg13873881) within the *AR*. **C** Relative *AR* mRNA levels (transcript variant corresponding to full length AR) and AR activity scores in the corresponding tissue samples, calculated based on Illumina Bead Chip arrays data as described in the methods section and analyzed in relation to the *AR* amplification status given in 3A. ***P < 0.001, # = DM-CpG

The *AR* mRNA overexpression observed in the M samples (Fig. 2B) corresponded to two transcript variants (coding for the full-length AR and its shorter AR45 variant, lacking the N-terminal domain). Accordingly, hypomethylation was observed at several *AR* promoter-associated CpGs in M compared to N and T samples, with the most pronounced CpGs being located close to the TSS200 and 5′-UTR regions (Fig. 3A). In order to examine if *AR* amplification, in addition to *AR* promoter hypomethylation, could be associated with the high *AR* mRNA levels observed, the *AR* copy numbers of the M samples were assessed and found to be amplified in 64% of the cases. Metastases with *AR* amplification showed the highest *AR* mRNA levels, but also M samples with a normal *AR* copy number had significantly higher expression levels than N and T samples (Fig. 3B). As seen in Fig. 3A, all but one of the untreated M samples had a normal *AR* copy number, while *AR* promoter hypomethylation could be observed already at that stage. Interestingly, there was no difference in AR activity in-between M samples with and without *AR* amplification, as determined by their score on a vector constructed from mRNA levels of genes involved in or regulated through canonical AR signaling (Fig. 3B). This indicates that AR activity within M samples is regulated by other means than by AR copy number, AR promoter methylation, and AR expression levels only.

### DNA promoter methylation associated with tumor androgen receptor activity and patient prognosis

**Fig. 4:**
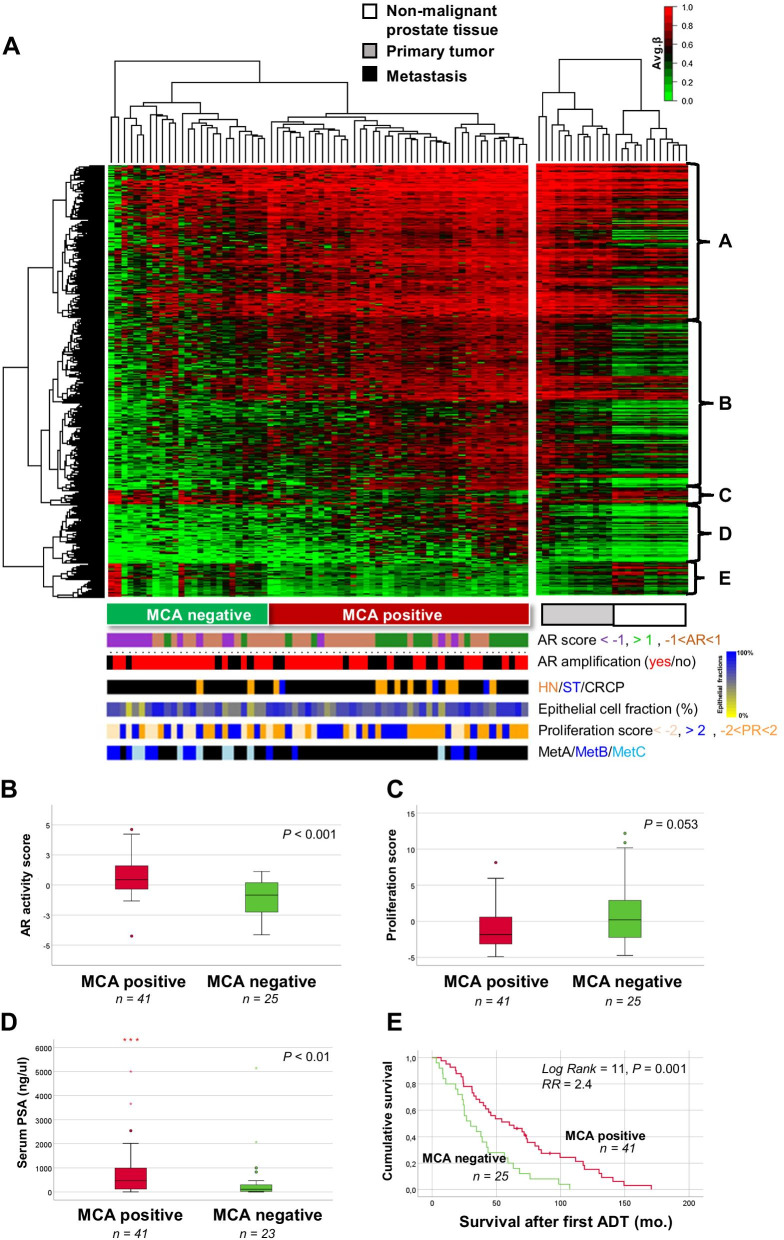
The promoter Methylation Classifier for Androgen receptor activity (MCA) signature in relation to tumor and patient characteristics. **A** Heat map showing the 4580 CpG sites in the MCA signature which correlated to AR activity score (corr > 0.4 or <  − 0.4, StDev > 0.15) in 66 bone metastases from PC. Primary tumors (*n* = 12) and non-malignant prostate tissue (*n* = 12) are shown as reference. MCA negative and MCA positive metastasis are marked in the figure. Colored bars show sample characteristics with respect to patient treatment status, tissue composition, MetA–C class, and *AR* copy number. Integrated expression analysis and enriched pathways for genes in cluster A–E are shown in Table 2. **B** AR activity scores of MCA positive and MCA negative bone metastasis. **C** Proliferation scores of the MCA positive and MCA negative bone metastasis samples. **D** Serum PSA levels of MCA positive and MCA negative patients with metastases. **E** Kaplan Meier survival analysis of MCA positive and MCA negative patients with metastases. The AR and proliferation scores are defined and calculated as described in the “Material and methods” section

To identify mCpGs related to the AR activity within PC metastases, and thus their potential AR dependence, we extracted data for all promoter CpG sites showing positive or negative correlation (correlation ≥ 0.4, StDev > 0.15) to the sample AR activity score. This Methylation Classifier for Androgen receptor activity (MCA) signature consisted of 2970 unique CpGs and separated the M samples into two main clusters named MCA negative and MCA positive (Fig. 4A). The frequency of HN, ST or CRPC samples did not significantly differ between MCA negative and positive metastasis, and neither did the frequency of *AR* amplified samples or the median epithelial cell fraction (Fig. 4A). Interestingly, the samples of the molecular metastasis subtype MetA were enriched in MCA positive metastasis, while MetB and MetC samples were enriched in MCA negative metastasis (*P* < 0.001, Chi-square test).

MCA positive metastasis showed hypomethylation and overexpression of genes associated with recurrent gene fusions in PC and AR activation and downstream signaling in PC (Fig. 4A, Table 2), indicating high AR dependence in those cases. In contrast, MCA negative showed hypermethylation and low expression of many genes related to AR activity and instead hypomethylation and high expression of genes associated with epithelial to mesenchymal transition (EMT), cytoskeletal remodeling, and immune response (Fig. 4A, Table 2). Accordingly, MCA negative metastases had significantly lower AR activity scores and a tendency of higher proliferation scores than MCA positive metastases (Fig. 4B, C). In addition, MCA negative patients showed significantly lower serum PSA levels and a worse prognosis after ADT than MCA positive patients (Fig. 4D, E). The median cancer-specific survival after ADT was 30 months for MCA negative patients compared to 60 months for MCA positive patients (*P* = 0.001, Fig. 4E). Analysis of cluster consistency showed an average silhouette width of 0.23 in the MCA positive and negative clusters (Additional file 3: Fig. S3).

**Table 2:** Genes corresponding to cluster A–E in Fig. 4A showing hyper- (A, B, D) or hypomethylation (C, E) in relation to AR activity in bone metastases, and functionally enriched pathways according to MetaCore analysis of genes with inverse correlation between CpG methylation and gene expression

Cluster	Genes	Enriched pathways
A	*ABCB9, ACOT11, ADAM28, ATHL1, AVIL, B3GNT5, BAALC, BATF, C14ORF173, C20ORF118, CCDC109B, CD300LF, CD38, CD5, CDC25B, CNPY4, CTLA4, E2F2, ENTPD3, FAM110B, GPR68, HCST, HEY1, HOXB4, HOXB5, HOXB6, INF2, KCNJ5, KIAA1274, KLHL2, KLK12, LIMS1, MAP1LC3A, MARCH3, MCOLN3, MDC1, MST1R, MXI1, NHS, NME2, NNT, NPL, OAS2, PARVB, PARVG, PAX6, PHF11, PIK3CD, PKM2, PLEKHO1, PPAP2C, PTGER1, RALA, RBM38, RBP1, ROD1, RPH3AL, RUNX2, SCNN1A, SCRN1, SDPR, SH2B3, SKA2, SLC43A3, SPC25, SPOCK2, SYK, TCF4, TCP11, TMEM105, TMEM176A, TNS3, TOX3, TRIM9, TYMS, UCHL1, WASPIP, VDR, VIM, YWHAH, ZBP1*	Development: TGF-beta-dependent induction of EMT via RhoA, PI3K and ILK Cytoskeleton remodeling: Reverse signaling by Ephrin-B, Integrin outside-in signaling Immune response: IL-15 signaling via JAK-STAT cascade, KLRK1 (NKG2D) signaling pathway, IL-2 signaling via JAK/ STAT, Role of DAP12 receptors in NK cells CHDI_Correlations from discovery data causal network SHH signaling in colorectal cancer
B	*ABP1, ACOT11, ACTB, ARHGEF2, BATF, C6ORF115, CCDC28B, CCR1, CD300LF, CD34, COL4A6, CX3CL1, ELF4, EMP1, EPHA2, FBXL10, FGR, FOXM1, GADD45A, GALE, GIMAP8, GMFG, HES6, HMGB3, HOXB3, HOXB4, HOXB6, IFI27, IL23A, IL2RB, IVNS1ABP, KLF13, KLHL6, LBX2, LRRN2, MAL, NCKAP1L, NDUFA4L2, NPAS2, NRM, NT5DC2, PAM, PARVG, PCK2, PIGR, PITX1, PPT2, RFXDC2, RNF34, SARDH, SDC1, SEL1L3, SERPINB1, SHANK3, SLC15A3, SLC35D3, SOX2, SPOCK2, STAT5A, SULT1A1, TAGLN2, TCF4, TIMP1, TMEM105, TMEM173, TOX3, TYROBP*	
D	*AGPS, BTG1, CD93, CENPV, GNAI2, LRFN4, NCAPG, NCF4, PRR7, PTK6, SC65, SDC1, SGOL1, TAGLN2, TGIF2, TMEM37, TNFAIP8L1, TSPAN4, UCP2*	
C	*ACSL3, BBS4, C10ORF81, CANT1, CREB3L4, CTDSP1, EHF, GREB1, MLPH, NDRG3, NEDD4L, NPAL3, NUP93, RPL37, SGMS1, STEAP2, SVIL*	Recurrent gene fusions in prostate cancer Transcription targets of androgen receptor involved in prostate cancer Androgen receptor activation and downstream signaling in prostate cancer TMPRSS2-ERG fusion in prostate cancer G-protein signaling_G-protein alpha-i signaling cascades Signal transduction_mTORC1 downstream signaling
E	*ATP6A, C19ORF48, CANT1, CLIP1, EHF, ELK4, GNL3, KIAA1244, NEDD4L, PEX11A, RAP1GAP, RGS10, SLC45A3, SPDF, STX19, SVIL, TMPRSS2, TRIM36, XPO6, ZNF280D*	

In Cox regression analysis, the MCA negative signature, proliferation score, and the MetB subtype were significantly associated with poor survival after ADT (Table 3). Multivariate Cox analysis indicated that the MCA signature provided prognostic information that was independent from the proliferation and subtype status, with an increased risk of 2.7 for patients being MCA negative (*P* = 0.0017) (Table 3). No significant associations were observed between patient prognosis and AR activity score, *AR* amplification status, serum PSA, or age (Table 3).

**Table 3:** Univariate and multivariate Cox regression survival analysis of MCA cluster and other characteristics of interest, in relation to cancer-specific survival after androgen-deprivation therapy

	*P*	*RR*	*95% CI*
Univariate			
MCA cluster (negative vs. positive, *n* = 25 and 41)	**0.0016**	2.4	1.4–4.1
AR score	0.35	0.9	0.8–1.1
Prol. score	**0.039**	1.1	1.0–1.1
AR (ampl. vs. WT, *n* = 24 and 42)	0.12	1.5	0.9–2.6
Age (at metastsis surgery)	0.42	1.0	1.0–1.0
Metastasis subtype^#^			
MetA (*n* = 46)		1.0	
MetB (*n* = 12)	**0.034**	2.0	1.1–3.9
MetC (*n* = 8)	0.95	0.98	0.45–2.1
Serum PSA (at metastasis surgery)	0.07	1.0	1.0–1.0
Multivariate			
MCA cluster (negative vs. positive, *n* = 25 and 41)	**0.0017**	2.7	1.5–5.1
Prol. score	0.89	0.99	0.90–1.1
Metastasis subtype		1.0	
MetA (*n* = 46)		1.6	0.52–5.2
MetB (*n* = 12)	0.40	0.58	0.25–1.3
MetC (*n* = 8)	0.20		

Variables with a P-value below 0.05 (shown in bold) in univariate survival analysis were taken further into multivariate analysis

^#^Metastasis subtypes according to [12]

## Discussion

This study integrates genome-wide promoter methylation data with gene expression data and describes consistent changes occurring during PC disease progression from non-malignant prostate epithelium to primary PC and further to bone metastatic disease. In metastatic samples, the study also specifically explores if promoter methylation levels are related to the sample AR activity, defined from transcript levels of genes recently described to differentiate clinically relevant molecular subtypes of PC bone metastases based on AR activity [9, 10, 12].

By pairwise comparisons of 12 tumor areas and adjacent non-malignant tissue isolated from radical prostatectomies, a pronounced hypermethylation was observed in primary PC. This is in line with a wealth of data, summarized in [15], suggesting not only that hypermethylation is an early event in PC tumorigenesis but also that it possibly could be used for diagnostic purpose. We observed hypermethylation of *GSTP1*, but also of other genes well known to be hypermethylated in PC, including *AOX1, APC, BARHL2, CCDC8, CDKN2A, CYP27A1, EFS, GRASP, HOXA3, HOXC11, HOXD3, KIT, NXK2-1, NXK2-5, PHOX2A, POU3F3, PTGS2, RARB, RHCG, SIX6, TBX15, TMEM106A, WNT2, and ZNF154* [15] (Additional file 4: Table S1). Interestingly, methylation changes in *APC, HOXD3,* and *PTGS2* have previously been suggested to provide prognostic information [27–29]. The low number of primary tumor samples analyzed in the current study did, however, not allow evaluation of DM-CpGs in relation to prognosis. Instead, analysis was focused on identifying general effects of methylation on changes in gene expression during PC development. Functional pathway analysis using Gene Set Enrichment Analysis indicated hypermethylation and downregulation of genes associated with muscle contraction, such as *ACTA, CNN, DES, TAGLN, and TPN,* normally expressed in smooth muscle cells [30], but also of genes involved in diverse developmental (e.g. *ANGPT1, STAT3, STAT5, TEK*) and cytoskeletal (e.g. *ANXA2, ANXA6, CD44, SVIL*) processes, as well as in regulation of proliferation (e.g. *FGF2, FGFR1, PIK3R1, PRKCA*) and cell adhesion (e.g. *CDH5, CDH23, PCDH18, STMN2*). While reduced cell adhesion is an important hallmark for carcinomas, many of the other functional effects suggested from promoter hypermethylation in PC may be biased not only by the more abundant stroma in the T samples, but also by its different cellular content. While the stroma in the normal prostate is mainly composed of smooth muscle cells, prostate tumors show a progressive loss of smooth muscle cells in the favor of cancer-associated fibroblasts [31]. Hypomethylation in primary PC was uncommon and not associated with specific functional processes, at least based on the literature summarized by MetaCore ontology software.

Most of the identified DM-CpGs observed between primary PC and non-malignant prostate tissue were present also in the metastatic tissue. In addition, a unique set of DM-CpGs were observed when going from primary PC to metastases. The large majority of these were hypomethylated and included sites in the *AR* promoter region*.* A detailed exploration showed a trend of demethylation at several CpGs in the *AR* promoter, including two alternative transcription start sites that correlated well with induced levels of both *AR* transcript 1 and 2 in the metastatic samples. High *AR* mRNA levels in CRPC have been previously associated with *AR* amplification [32–36], while methylation as a regulator of *AR* expression is less well described and studies have also shown inconsistent results [16, 37–40]. Our results are in line with recent findings by Zaho and co-workers demonstrating *AR* promoter hypomethylation in CRPC [16], but also add important knowledge by showing hypomethylation in previously un-treated metastasis samples suggesting that *AR* demethylation may be an earlier cause to *AR* induction in PC metastases than *AR* amplification, as *AR* amplification is generally not observed prior to ADT. Importantly, neither the large variance in *AR* promoter methylation levels observed among the metastatic samples nor their *AR* amplification status showed any clear relationship to the tumor sample AR activity.

In an attempt to study universal promoter methylation in relation to AR activity within individual metastasis samples, each sample was given an AR activity score related to its expression levels of genes involved in or regulated by canonical AR signaling, and mCpGs positively or negatively correlated to this score were taken further for functional exploration. A Methylation Classifier for Androgen receptor activity (MCA) signature was identified and used to classify the bone metastasis samples. Taken together, our results suggested that low promoter methylation levels of certain AR-regulated genes (i.e. *SLC45A3, STEAP2, ELK4* and *TMPRSS2*) may contribute to the AR-driven tumor phenotype seen in the majority of CRPC patients with metastatic disease [2–12], while high promoter methylation of the same genes may contribute to the development of less AR-dependent CRPC, commonly referred to as AR-indifferent, small-cell or neuroendocrine-like CRPC [41]. It is of importance to note, though, that histological evaluation did not confirm any enrichment of a neuroendocrine-like phenotype in samples of the MCA negative compared to the positive cluster. This was concluded from reviewing the results from immunohistochemical analysis of the neuroendocrine marker chromogranin A, previously assessed in 60 of the 66 MCA-classified samples [12] (data not shown). Neither could the MCA class be predicted from immunohistochemical analysis of the AR protein level [12] (*n* = 63, data not shown).

Samples of the metastasis subtype MetA previously described by us as AR-driven [12] primarily showed hypomethylation and high expression of canonically AR-regulated genes, while samples of the less AR-driven subtypes MetB and MetC were enriched among samples with an MCA negative signature (showing hypermethylation and low expression of many AR targeting genes). Indirectly, our result thus implies promoter methylation as a possible mechanism behind the low AR activity defining metastasis subtypes MetB and MetC. Accordingly, patients with MCA positive signature had higher serum PSA levels and a better prognosis than the smaller group of MCA negative patients, probably due to more differentiated, less proliferative tumor cells being more AR dependent and thereby showing a better response to AR targeting therapies, similar to what previously have been described for MetA in comparison to MetB–C [12]. Notably, the MCA signature provided strong information related to patient prognosis after ADT, with MCA positive patients showing 2-times longer median survival than MCA negative patients, resulting in a 5-year cancer-specific survival of 50 and 20% respectively. Despite the obvious clinical relevance of separating metastasis samples into MCA positive or negative cases, the clusters showed variable consistencies according to the silhouette analysis. This might originate from the procedure of selecting all mCpG sites for analysis that correlated to the tumor AR activity on a continuous scale, i.e. the classifier was not originally built to dichotomize samples into two groups.

As MCA negative metastases with low AR activity scores were specified by hypermethylation of AR-regulated genes, it is tempting to speculate that they could be sensitized for AR-directed therapies by treatment with demethylating agents, such as have been shown possible by inhibitors to DNA methyl transferase 1 in different experimental systems for CRPC [42, 43]. In a recent paper, Xiao and co-workers furthermore identified the histone lysine-*N*-methyltransferase EZH2 as a possible epigenetic regulator of tumor response to AR-targeting therapy and demonstrated possibilities with its inhibition in the treatment of CRPC [44].

While the methylation levels of a relatively small set of genes were directly associated with the AR activity observed in PC bone metastases, the methylation level of a much larger gene set seemed involved in regulating diverse processes such as EMT, cytoskeletal remodeling, and immune responses, all standing in an inverse correlation to tumor AR activity.

In conclusion, this study describes general patterns of gene promoter methylation during PC disease progression, and specifically identifies a novel promoter MCA signature that is associated with canonical AR activity in PC bone metastases and to patient prognosis after ADT. Based on this methylation signature, patients with PC bone metastases could be stratified into one of two patient groups; MCA positive patients being likely to respond to ADT and possibly also other AR targeting therapies or MCA negative patients being less responsive to AR targeting therapies, but possibly suitable for treatment with AR inhibitors in combination with epigenetic modulators (currently in clinical trials, https://clinicaltrials.gov). The next step will be to verify the suggested therapy-predictive value of the MCA signature. This could be retrospectively done by analyzing methylation signatures in circulating tumor DNA collected from patients prior to treatment for metastatic PC, similarly to what is described in a recent paper by Wu et al. [45]. For clinical implementation, however, a stable non-cluster-based classifier need to be developed. The overall cause to the two methylation types observed among PC bone metastases also remains to be identified, as it could give important implications for future developments of novel treatment strategies.

## Supplementary Information

**Additional file 1: Fig. S1.** Analysis of 59 built-in SNP on the HumanMethylation EPIC array to confirm identity of multiple samples taken from the same individual (N and T tissue).

**Additional file 2: Fig. S2.** Schematic flowchart of the pre-processing steps of the HumanMethylation EPIC arrays.

**Additional file 3: Fig. S3.** Silhouette analysis showing the cluster consistencies of the MCA positive and negative clusters in Figure 4A.

**Additional file 4:** **Table S1.** Differetially methylated CpGs in promoter regions of genes in primary prostate tumor tissue (T) compared to adjacent non-malignant prostate tissue (N), their correlations to corresponding gene expression levels, and the fold change in gene expression levels between sample groups.

**Additional file 5:**  **Table S2.** Top 10 enriched process networks in localized prostate tumor tissue (T) compared to adjacent non-malignant prostate tissue (N) or in prostate cancer bone metastases (M) compared to localized tumors.

**Additional file 6:** **Table S3.** Differetially methylated CpGs in promoter regions of genes in bone metastases (M) compared to localized prostate tumors (T), their correlations to corresponding gene expression levels, and the fold change in gene expression levels between sample groups.

**Additional file 7:** **Table S4.** CpG sites of the AR gene shown in Figure 3A.

## Data Availability

The dataset generated (GSE174613) and analyzed (GSE29650 and GSE101607) during the current study are available at https://www.ncbi.nlm.nih.gov/geo/.

## Abbreviations

AR: Androgen receptor
ADT: Androgen-deprivation therapy
*β*-value: Methylation level
CNV: Copy number variation
CRPC: Castration-resistant prostate cancer
DM: Differentially methylated
EMT: Epithelial to mesenchymal transition
HN: Hormone-naïve
M: Metastasis tissue
MCA: Methylation classifier for androgen receptor activity
mCpGs: Methylation levels (*β* values) of CpG sites
MetA–C: Metastasis subtype A–C
meQTL: Methylation quantitative trait loci
N: Non-malignant prostate tumor tissue
PC: Prostate cancer
PCA: Principal component analysis
PSA: Prostate specific antigen
SNP: Single nucleotide polymorphism
ST: Short-term castrated
T: Malignant prostate tumor tissue
TSS: Transcription start site
