# Supplementary figures and images for: A novel DNA methylation signature is associated with androgen receptor activity and patient prognosis in bone metastatic prostate cancer
Source: Clin Epigenetics. 2021 Jun 30;13:133. doi: 10.1186/s13148-021-01119-0 (PMC8244194; doi:10.1186/s13148-021-01119-0)

Figure S1

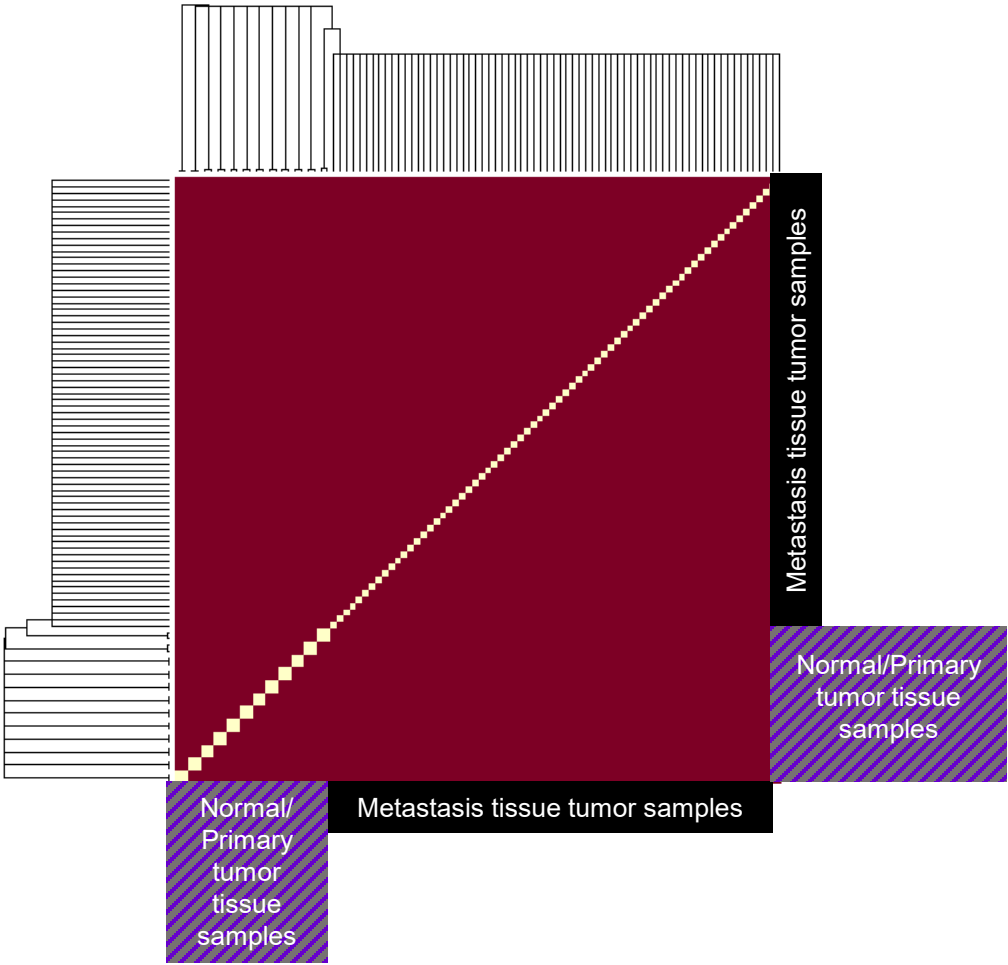

Supplement: Supplementary file 1 — Additional file 1: Fig. S1. Analysis of 59 built-in SNP on the HumanMethylation EPIC array to confirm identity of multiple samples taken from the same individual (N and T tissue). [file 13148_2021_1119_MOESM1_ESM.pdf]

## Figure S2

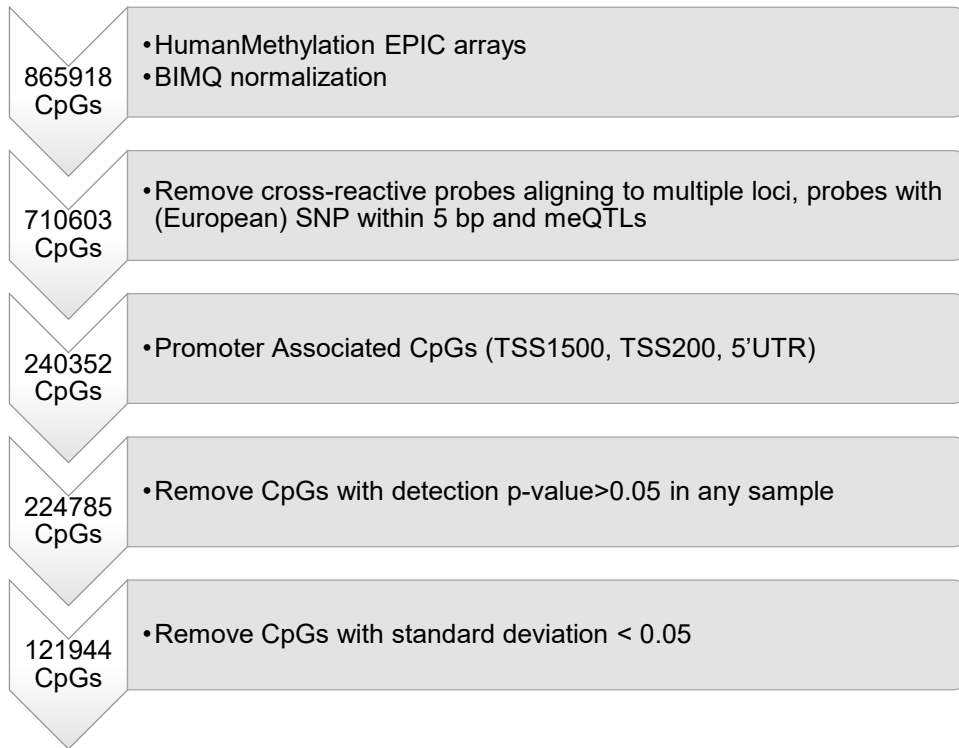

Supplement: Supplementary file 2 — Additional file 2: Fig. S2. Schematic flowchart of the pre-processing steps of the HumanMethylation EPIC arrays. [file 13148_2021_1119_MOESM2_ESM.pdf]

Figure S3

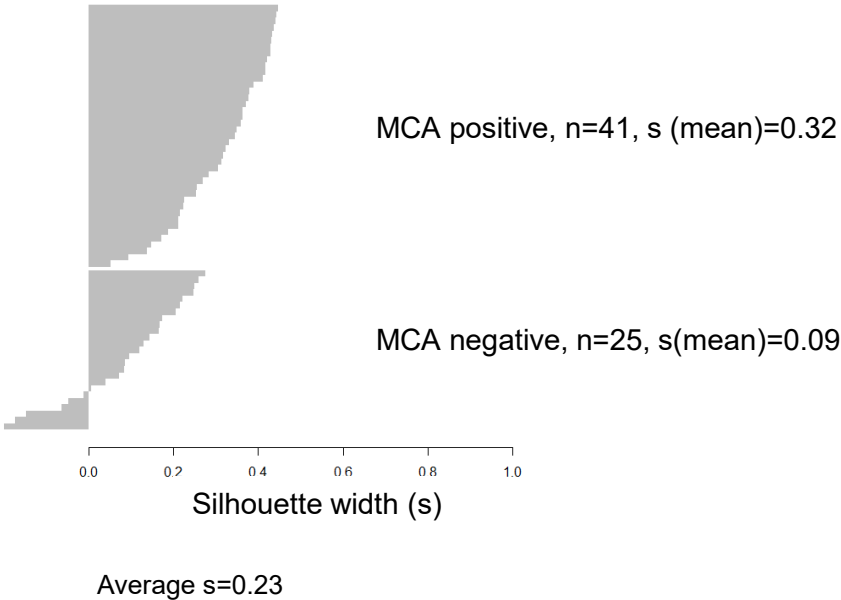

Supplement: Supplementary file 3 — Additional file 3: Fig. S3. Silhouette analysis showing the cluster consistencies of the MCA positive and negative clusters in Figure 4A. [file 13148_2021_1119_MOESM3_ESM.pdf]
