# Supplementary material for: A novel DNA methylation signature is associated with androgen receptor activity and patient prognosis in bone metastatic prostate cancer
Source: Clin Epigenetics. 2021 Jun 30;13:133. doi: 10.1186/s13148-021-01119-0 (PMC8244194; doi:10.1186/s13148-021-01119-0)
Supplement: Supplementary file 5 — Additional file 5: Table S2. Top 10 enriched process networks in localized prostate tumor tissue (T) compared to adjacent non-malignant prostate tissue (N) or in prostate cancer bone metastases (M) compared to localized tumors. [file 13148_2021_1119_MOESM5_ESM.docx]

**Table S2.** Top 10 enriched process networks in localized prostate tumor tissue (T) compared to adjacent non-malignant prostate tissue (N) or in prostate cancer bone metastases (M) compared to localized tumors.

| **Comparison**  **(Genes in analysis)** | **Hypermethylated promoter CpG, decreased gene expression** | **Hypomethylated promoter CpG, increased gene expression** |
| --- | --- | --- |
| T vs. N  (442, 67) | - *Muscle contraction* - *Development: i) Regulation of angiogenesis, ii) Blood vessel morphogenesis, iii) Skeletal muscle development, iv) Hemopoiesis, Erythropoietin pathway* - *Cytoskeleton: Actin filaments* - *Proliferation: Positive regulation cell proliferation* - *Cell adhesion: i) Cell-matrix interactions, ii) Cadherins* - *Cell cycle: G1-S Growth factor regulation* | - *No consensus* |
| M vs. T  (23, 70) | - *Apoptosis: Anti-apoptosis mediated by external signals via NF-kB* | - *No consensus* |
